# Statistical Analysis of *Hie* (Cold Sensation) and *Hiesho* (Cold Disorder) in Kampo Clinic

**DOI:** 10.1155/2013/398458

**Published:** 2013-12-30

**Authors:** Tetsuhiro Yoshino, Kotoe Katayama, Kaori Munakata, Yuko Horiba, Rui Yamaguchi, Seiya Imoto, Satoru Miyano, Kenji Watanabe

**Affiliations:** ^1^Center for Kampo Medicine, Keio University School of Medicine, 35 Shinanomachi, Shinjuku-ku, Tokyo 160-8582, Japan; ^2^Human Genome Center, The Institute of Medical Science, The University of Tokyo, 4-6-1 Shirokanedai, Minato-ku, Tokyo 108-8639, Japan; ^3^Faculty of Environmental and Information Study, Keio University, 5322 Endo, Fujisawa, Kanagawa 252-0882, Japan; ^4^Center for Preventive Medicine, Keio University School of Medicine, 35 Shinanomachi, Shinjuku-ku, Tokyo 160-8582, Japan

## Abstract

A cold sensation (*hie*) is common in Japanese women and is an important treatment target in Kampo medicine. Physicians diagnose patients as having *hiesho* (cold disorder) when *hie* disturbs their daily activity. However, differences between *hie* and *hiesho* in men and women are not well described. *Hie* can be of three types depending on body part where patients feel *hie*. We aimed to clarify the characteristics of patients with *hie* and *hiesho* by analyzing data from new patients seen at the Kampo Clinic at Keio University Hospital between 2008 and 2013. We collected information about patients' subjective symptoms and their severity using visual analogue scales. Of 4,016 new patients, 2,344 complained about *hie* and 524 of those were diagnosed with *hiesho*. *Hie* was most common in legs/feet and combined with hands or lower back, rather than the whole body. Almost 30% of patients with *hie* felt upper body heat symptoms like hot flushes. Cold sensation was stronger in *hiesho* than *non-hiesho* patients. Patients with *hie* had more complaints. Men with *hiesho* had the same distribution of *hie* and had symptoms similar to women. The results of our study may increase awareness of *hiesho* and help doctors treat *hie* and other symptoms.

## 1. Introduction

**Figure 1:**
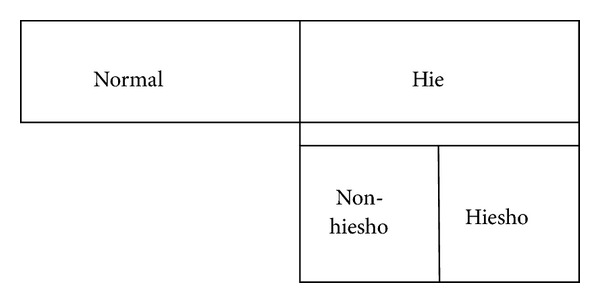
*Hie* and *hiesho*. In Japan *hie* (cold sensation) and *hiesho* (cold disorder) are different terms. While *hie* is the term used to describe the subjective, uncomfortable feeling of coldness, *hiesho* is the diagnosis given by physician to patients with cold sensation disturbing their daily living. Therefore, the first distinction is between normal and *hie* group. The *hie* group is subdivided into *hiesho* and *non-hiesho*.

In Japan, *hie* (cold sensation) and *hiesho* (cold disorder) are different terms. While *hie* is used to describe the subjective, uncomfortable feeling of coldness, *hiesho* is the diagnosis given by physicians to patients with cold sensations that disturb their daily living. Therefore, the first distinction to make is one between normal and *hie* groups. Those who experience *hie* can further be subdivided into *hiesho* and *non-hiesho* categories (Figure 1).

*Hiesho* is the most common diagnosis given in Japanese Kampo clinics [1]. In Japanese Kampo medicine, *hiesho* is treated as a unique pathological condition. In contrast, cold sensation is only one of many symptoms asked about in a review of systems in Western medicine. One definition of *hiesho* for diagnosis is an “abnormal, subjective sensitivity to coldness in the lower back, the extremities, other localized regions of the body, or the whole body despite ambient temperatures. It lasts throughout the year for most patients, and disturbs their daily living” [2].

*Hie* as a subjective symptom is common in Japanese people [1] and is more common in women [3]. However, the epidemiology of this symptom is not clear in Western people. One report comparing Japanese with Brazilians indicated that 57% of Brazilian pregnant women were aware of cold sensations [4]. We think it may be common symptom in other populations as well. In 1987, Kondo and Okamura reported demographic data of 318 Japanese women with *hie* but had no data for men [5]. They reported that *hie* accompanied other uncomfortable symptoms such as shoulder stiffness, constipation, lumbago, fatigue, and hot flushes. In Kampo medicine, treatments not only target *hie*, but also these accompanied symptoms. Subsequently, there are many Kampo formulas for treating *hiesho*.

*Hie* has been categorized into three types based on the body part where the symptoms are experienced. We assume different pathophysiology for each type. The first type of *hie* is a general type due to decreased heat production from a loss of muscle volume or decreased basal metabolism. The second type of *hie* is peripheral, due to a disturbance of heat distribution related to decreased peripheral blood flow. The third type of *hie* is upper body heat-lower body coldness with associated vasomotor abnormalities. However, epidemiological information regarding these classifications are unknown.

Keio University first introduced a browser-based questionnaire in 2008 that collects patient's subjective symptoms and changes in symptom severity via visual analogue scales (VAS), life styles, Western and Kampo diagnoses, and prescribed Kampo formulas.

Here, we report results from the analysis of data from male and female patients and attempt to clarify the characteristics associated with *hie* and *hiesho*. We especially focus on classification of *hie* and accompanied symptoms because this information is important for considering the pathophysiology of *hie* and the appropriate Kampo formulas for treating patients with *hiesho*.

## 2. Methods

### 2.1. Patient Enrollment

Patients who made their first visit to the Kampo Clinic at Keio University Hospital between May 2008 and March 2013 were included from this study. Exclusion criteria were unwillingness to enter the study and missing data regarding age and/or sex. Patients who answered only about their lifestyle or who were diagnosed as having *hiesho* but did not answer regarding the part of the body where they felt *hie* were excluded. All registered patients provided written informed consent.

### 2.2. Patient Grouping

In this analysis, we divided patients into three groups: patients with *hie* with a diagnosis of *hiesho* (*hiesho* group), patients with *hie* without a diagnosis of *hiesho* (*non-hiesho* group), and patients without *hie* (Normal group). Our dataset did not include information about how physicians diagnosed patients with *hiesho* (Figure 1).

### 2.3. Assessment of Subjective Symptoms

We collected information about patients' subjective symptoms using a 128-question binary questionnaire (Table 1). Among these 128 questions, 106 also had VAS when patients answered yes on the binary questionnaire. The VAS was a horizontal line, 100 mm in length, where the left-most side (0 mm) represented no symptoms and right-most side (100 mm) represented the severest symptoms. To normalize within each patient, we divided each patient's VAS by the maximum VAS possible. This is because VAS scores were different from patient to patient. In other words, each patient's original VAS values ranged from 0 to 100 but were transformed to 0 to 1 for easier comparison.

### 2.4. Between Group Comparisons

We focused on symptoms directory related to *hie* to clarify the differences between the *hiesho* and *non-hiesho* groups. Here, we choose six symptoms from the directory related to *hie*: *hie* of the whole body, *hie* of the hands, *hie* of legs/feet, *hie* of the lower back, cold intolerance, and tendency to get frostbite.

We also analyzed body part combinations where patients felt *hie* and five heat-related symptoms to get epidemiological information regarding *hie* classification. The five heat-related symptoms were as follows: heat intolerance, hot flush, heat sensation of the face, heat sensation of the hands, and heat sensation of legs/feet.

Finally, we focused on accompanying symptoms and compared men and women to clarify differences between these groups.

### 2.5. Statistical Analysis

All statistical analyses were conducted using R software, version 2.15.2 (The R Foundation for Statistical Computing; October 26, 2012). Characteristics were compared using Wilcoxon's rank sum test, two-sample *t*-test, and test for equal proportions. We used Wilcoxon's rank sum test to compare the VAS of *hie* because normality did not hold. We used a significant level of 5% for all tests.

## 3. Results

### 3.1. Participant Information

Participants included 4,057 registered patients, 41 of whom were excluded because of missing values (one due to missing age, 19 failed to report anything regarding subjective symptoms, and 21 were missing data on the part of the body where they felt *hie* in spite of a diagnosis of *hiesho*). We used data from 4,016 patients in this analysis, including 2,344 patients with *hie*, and 524 of those who were diagnosed as having *hiesho*.

### 3.2. Age and Sex

**Figure 2:**
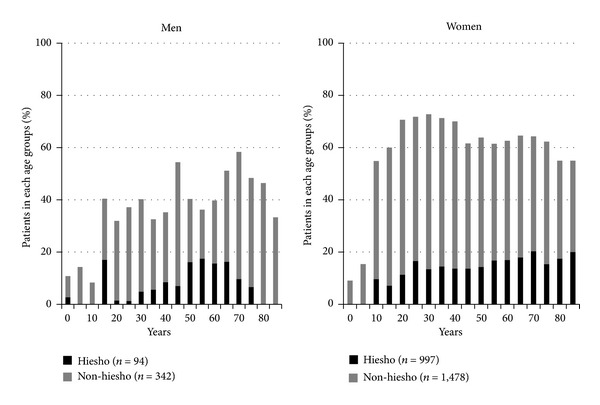
Rate of *non-hiesho* and *hiesho* groups in each age group. *Hie* (cold sensation) and *hiesho* (cold disorder) were uncommon in children, but almost similarly present among young and old patients. We also can see that *hie* and *hiesho* were more common in women.

We compared age and sex of patients with *hie* with the diagnosis of *hiesho* (*hiesho* group, *n* = 524) and patients with *hie* but no diagnosis of *hiesho* (*non-hiesho* group, *n* = 1,820) to patients without *hie* (Normal group, *n* = 1,672). The mean age was 51.6 ± 1.5 years old for members of the *hiesho* group, 47.1 ± 0.8 years old for the *non-hiesho* group, and 46.2 ± 1.0 years old for the Normal group. Participant mean age in the *hiesho* group was significantly higher than the *non-hiesho* and Normal groups according to results of a *t*-test. The number of patients in each group who fell within each age group is shown in Figure 2. *Hie* and *hiesho* were uncommon in children and rates were similar for young and old patients.

With regard to sex, there were 94 men and 430 women (percentage of women: 82.1%) in the *hiesho* group, 342 men and 1,478 women (percentage of women: 81.2%) in the *non-hiesho* group, and 675 men and 997 women (percentage of women: 59.6%) in the Normal group. A test for equal proportions showed significantly more women in both the *hiesho* and *non-hiesho* groups than in the Normal group.

### 3.3. Differences between *Hiesho* and *Non-Hiesho* Groups

**Figure 3:**
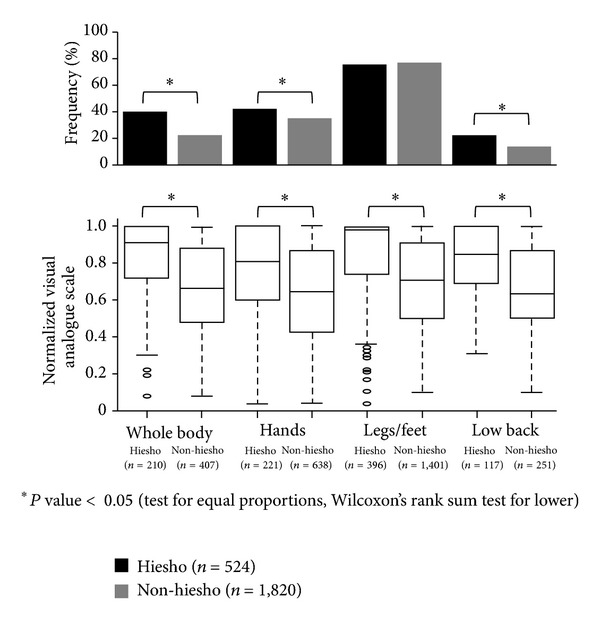
Frequency and severity of *hie* (cold sensation) by affected areas. The frequencies of binary answers of four parts of the body where patients felt *hie* were significantly higher in *hiesho* group except for *hie* of legs/feet as per the test for equal proportions (upper figure). Normalized visual analogue scales of *hie* of each body part of *hiesho* group were compared to *non-hiesho* group by Wilcoxon's rank sum test. *Hie* in every part of the body was significantly worse in *hiesho* group (lower figure).

We compared the location where *hie* symptoms occurred between the three groups. The frequencies of binary answers for the four parts of the body where patients felt *hie* for *hiesho* and *non-hiesho* groups are as follow: *hie* of the whole body: *hiesho* 40.1%, *non-hiesho* 22.4%; *hie* of the hands: *hiesho* 42.2%, *non-hiesho* 35.1%; *hie* of the legs/feet: *hiesho* 75.6%, *non-hiesho* 77.0%; and *hie* of the lower back: *hiesho* 22.3%, *non-hiesho* 13.8%. Except for legs/feet, the frequencies of *hie* were significantly higher for the *hiesho* group based on results of the test for equal proportions (Figure 3 upper). There were no clear differences seen regarding the distribution of *hie* based on patient age or sex.

The frequencies of binary answers of the other two *hie* related symptoms for all three groups are as follows: cold intolerance: *hiesho* 77.7%, *non-hiesho* 58.0%, and Normal 16.1%; and tendency to get frostbite: *hiesho* 10.7%, *non-hiesho* 6.3%, and Normal 1.5%. The frequencies of binary answers of the two symptoms were significantly higher in the *hiesho* group than the *non-hiesho* group, which were both higher than Normal group as determined by the test for equal proportions. We also compared the differences of VAS scores for *hie* of each body part for members of the *hiesho* and *non-hiesho* groups using Wilcoxon's rank sum test. For every part of the body, *hie* was significantly worse for members of the *hiesho* group (Figure 3 lower). In the same way, VAS values for cold intolerance in the *hiesho* group also were higher than those in the *non-hiesho* group, which were higher than those in the Normal group.

### 3.4. Body Part Combinations of *Hie* and Frequencies of Heat-Related Symptoms

**Figure 4:**
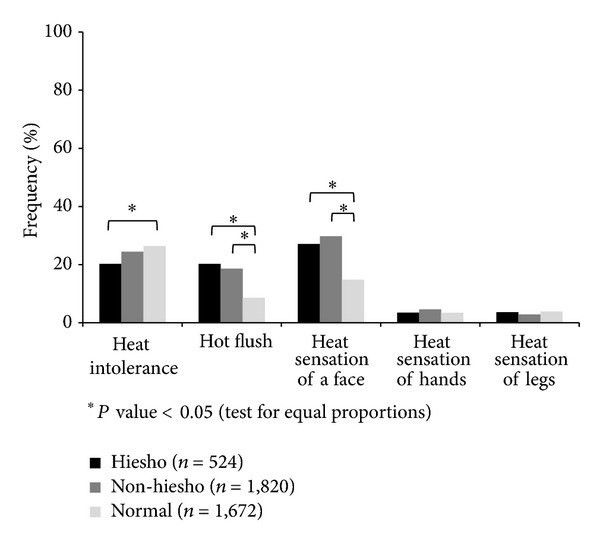
Heat symptoms for *non-hiesho* and *hiesho* groups. The frequencies of binary answers about upper body heat symptoms, such as hot flushes and heat sensation of the face, were significantly common in patients with *hie* (cold sensation). All data were compared using the test for equal proportions.

We also analyzed body part combinations where patients felt *hie* and frequencies of heat-related symptoms to obtain epidemiological information regarding the classification of *hie*. Regarding body part combination of *hie* symptoms for patients in the *hiesho* and *non-hiesho* group (*n* = 2,344), 722 patients felt *hie* in both their hands and legs/feet among 859 patients who felt *hie* in their hands; that is, 84.2% of patients who felt *hie* in their hands also felt *hie* in legs/feet. Similarly, among 368 patients who felt *hie* in their lower back, 286 (77.7%) also felt *hie* in their legs/feet. In contrast, among the 617 patients who felt *hie* throughout their whole body, 265 (43.0%) also felt *hie* in their legs/feet and this ratio was significantly lower than the former two as determined by the test for equal proportions (Table 2). We also focused on five heat-related symptoms for the three groups: heat intolerance: *hiesho* 20.2%, *non-hiesho* 24.5%, and Normal 26.4%; hot flushes: *hiesho* 20.2%, *non-hiesho* 18.6%, and Normal 8.6%; heat sensation in the face: *hiesho* 27.1%, *non-hiesho* 29.8%, and Normal 14.8%; heat sensation in the hands: *hiesho* 3.4%, *non-hiesho* 4.6%, and Normal 3.4%; and heat sensation of the legs/feet: *hiesho* 3.6%, *non-hiesho* 2.9%, and Normal 3.9% (Figure 4). We did not separate these groups by sex, as men in the *hiesho* and *non-hiesho* groups had higher frequencies of hot flush or heat sensation of the face the same as women in *hiesho* group or *non-hiesho* group. The frequency of heat intolerance was significantly lower for the *hiesho* group compared to Normal group. In contrast, hot flush and heat sensation of the face were significantly more frequent for members of the *hiesho* and *non-hiesho* groups compared to the Normal group as indicated by the test for equal proportions.

### 3.5. Accompanying Symptoms

We also compared accompanying symptoms in members of the three groups. Of 122 symptoms, after removing the 6 *hie* related symptoms, the mean number of subjective symptoms reported was 22.9 ± 1.0 for members of the *hiesho* group, 24.5 ± 0.6 for the *non-hiesho* group, and 15.8 ± 0.5 for the Normal group (Table 3). The mean number of subjective symptoms for both the *hiesho* and *non-hiesho* groups was significantly higher compared to the Normal group as indicated by the *t*-test. We sorted symptoms by reporting frequency for the *hiesho* group. The top 10 common symptoms were as follows: shoulder stiffness, easily fatigued, neck stiffness, eyestrain, depressed mood, constipation, upper back stiffness, dry skin, flatulence, and forgetfulness. In women, menstrual pain also was common. The ranking of these symptoms was almost the same for members of both sexes and all three groups.

## 4. Discussion

Kampo physicians diagnose patients as having *hiesho* (cold disorder), when *hie* (cold sensation) and its associated symptoms cause disturbance in daily living. In Japanese Kampo medicine, *hiesho* is treated as a unique pathological condition and there are many Kampo formulas to treat it. When choosing Kampo formulas, the part of the body where *hie* and its accompanied symptoms are felt is important. This is why the present study has focused on the classification of *hie* and its comorbid symptoms.

Fundamental parts of our dataset were consistent with previous reports and supported the generalizability of our data, despite our population being recruited from a Kampo clinic. It has been reported that the subjective symptom of *hie* was common in Japanese people and a diagnosis of *hiesho* was common in Japanese Kampo clinics [1, 6]. Consistent with these past reports, around 60% of patients in our study reported subjective feelings of *hie*, and *hiesho* was one of the most common diagnoses in the Kampo medicine clinic where our study was conducted. It also has been reported that *hie* and *hiesho* are common in women [3], which is consistent with results of the present study. The frequency of patients in our study who reported experiencing *hie* in their extremities was also consistent with the results of past studies from an obstetrics-and-gynecology clinic in Japan [7] and on working women in Japan [5]. Ushiroyama mentioned that women developed *hie* because of the existence of their pelvic organs, which affected peripheral blood flow to the legs/feet and lower back [7]. Women's pelvic organs develop after puberty and may consume blood flow of lower body. However, according to our research, the legs/feet were the most common parts of the body affected by *hie* for both men and women of all age groups. Thus, explanations regarding the effect of pelvic organs do not help us understand lower body *hie* in men and postmenopausal women.

We found that patients diagnosed as having *hiesho* reported more severe *hie* symptoms. The frequencies of *hie* of the whole body, hands, and lower back as well as reports of cold intolerance and a tendency to get frostbite were higher in the *hiesho* compared to *non-hiesho* group. Furthermore, patients in the *hiesho* group were more likely to have high VAS scores regarding *hie* for any body part and cold intolerance compared to their *non-hiesho* counterparts. There were no other symptoms for which patients in the *hiesho* group had higher VAS scores than those in the *non-hiesho* group. In addition, hypothyroidism was significantly more common in the *hiesho* than *non-hiesho* group (2.5% versus 0.7%); however, most patients in the *hiesho* group did not have organic diseases that might cause *hie* (data not shown). It might be important for us to not only treat *hiesho*, but also to study organic diseases that can cause *hie*, especially in members of the *hiesho* group.

One classification categorizes *hie* into the three as per the areas of the body where people report experiencing it: general, peripheral, and upper body heat-lower body coldness. At the 51st annual meeting of the Japan Society for Oriental Medicine, Kako Watanabe et al. reported the efficacy of the cold-water challenge test to divide *hie* into these three types (not published). They put patients' hands into cold water at 4°C for 30 seconds and measured blood flow recovery. Patients with decreased metabolism complained of whole body *hie* after the cold-water challenge despite normal blood flow recovery, and patients with disturbed peripheral blood flow could not recover blood flow after the cold-water challenge test. In addition, patients with upper body heat-lower body coldness recovered blood flow with fluctuation due to autonomic imbalance. In Kampo theory, the pathophysiology of these three types of *hiesho* has been explained as qi deficiency, blood stagnation, and qi counterflow.

Our results support this classification of *hie*. We observed that many patients who report feeling *hie* in their hands or lower back also felt it in their legs/feet, and these combinations were far more frequent than the combination of whole body and legs/feet. The result supports the first two types of *hie* (general and peripheral). Our results also suggest that the peripheral type might be further subdivided by the type of extremity (e.g., narrowly defined extremity type, which affected hands and legs/feet, and lower body type, which affected the lower back and legs/feet). The general type of *hie* is thought to be related to a loss of heat production from decreased muscle volume and/or basal metabolism, and peripheral *hie* may be due to disturbances in heat distribution due to blood stagnation. We also found that around 20–30% of patients with *hie* felt upper body heat sensations such as hot flushes and heat sensation of the face, and these symptoms were significantly more common in patients with *hie*. This supports the existence of upper body heat-lower body coldness. This type of *hie* may be related to a kind of autonomic imbalance that causes vasomotor disturbances.

We assume representative Western diagnosis for these three types of *hie*. First, one of the organic diseases that causes general *hie* is hypothyroidism. Due to low metabolism, patients complain about feeling cold or cold intolerance, which sometimes may be comorbid with objectively cool peripheral extremities [8]. Based on a randomized crossover trial, thyroxin did not appear effective for patients with normal thyroid function tests and symptoms of hypothyroidism including intolerance to cold [9]. Next, one of the organic diseases that causes peripheral *hie* is peripheral arterial disease due to arteriosclerosis [10]. It is a good adaptation of Western intervention when patients feel acute coldness with resting pain in their foot and toes by critical limb ischemia such as occlusion of an artery where blood flow cannot accommodate basal nutritional needs of the tissues [11]. However, the majority of patients feel chronic cold sensations in their legs/feet without gait disturbances and it is difficult to treat such patients in Western medicine. Finally, one of the organic diseases that causes upper body heat-lower body coldness is perimenopausal disturbance. Hot flushes with lower extremity coldness due to vasomotor disturbance is common for peri- or postmenopausal women [12]. Treatment options are limited for some patients due to side effects of hormone replacement therapy. Kampo medicine may be one treatment option for such patients and we try to apply the appropriate Kampo formulas.

Our data supported that patients with *hie* experienced many uncomfortable symptoms, which may be aggravated by *hie*. It has been reported that women with *hie* and *hiesho* experienced other uncomfortable symptoms such as shoulder stiffness, constipation, lumbago, fatigue, hot flush, headache, and edema in the leg [3, 5]. Our findings support these results for both the sexes; menstrual pain often was found in women with *hie*. Thus, treatment of *hie* may lead to not only its improvement, but also to the improvement of other symptoms. However, the number of symptoms experienced by patients might affect our results, as patients with *hie* reported about 10 more symptoms than those without *hie*. This suggests that patients with *hie* had 1.6–1.8 times more symptoms than patients without *hie*. We also can assume that *hie* is an indicator of patients with many symptoms. Thus, we may obtain more information by segregating patients with *hie* according to their comorbid symptoms.

## 5. Conclusion

The present study is important because it clarifies some of the epidemiological characteristics of patients with *hie* and *hiesho*. Specifically, we have learned the following. (1) *hiesho* patients are those who suffer from severe *hie*. (2) Patients with *hie* may be classified roughly into three types. (3) Patients with *hie* experience many comorbid symptoms. (4) Men and women with *hiesho* have almost the same distribution of *hie* and its associated symptoms. Appropriate treatment options for *hiesho* are not available in Western medicine. Therefore, if we are more aware of *hiesho*, we can use Kampo formulas to treat not only the patients' *hie*, but their comorbid symptoms as well.

## Figures and Tables

**Table 1:** Questionnaire items.

No.	Binary questions	No.	Questions with visual analogue scales	No.	Questions with visual analogue scales

1	Appetite loss	1	Difficulty falling asleep	54	Heat hands
	Good appetite	2	Arousal during sleep	55	Heat legs
2	Slow speed of the meal	3	Early-morning awakening	56	Edema face
	Fast speed of the meal	4	Difficulty urinating	57	Edema hands
3	I dream frequently	5	Urination pain	58	Edema legs
4	Single dose of urine large	6	Urine leakage	59	Headache
	Single dose of urine low	7	Enuresis	60	Sluggishness
5	Hard stool	8	Diarrhea	61	Vertigo
6	Small and round stool	9	Hemorrhoid	62	Lightheadedness
7	Soft stool	10	Anal prolapse	63	Dandruff
8	Hard to stool	11	Bloody stool	64	Hair loss
9	Taking laxatives	12	Depressed mood	65	Decreased visual acuity
10	White nasal discharge	13	Forgetfulness	66	Eyestrain
	Yellow nasal discharge	14	Irritated	67	Blurred vision
11	White sputum	15	Dry skin	68	Bleary eyes
	Yellow sputum	16	Itchy skin	69	Dark circles under eyes
12	Abdominal pain fasting	17	Acne	70	Sneezing
13	Abdominal pain after eating	18	Blot	71	Post nasal drip
14	Abdominal pain at upper	19	Urticaria	72	Stuffy nose
15	Abdominal pain at lower	20	Wart	73	Nosebleed
16	Heavy menstrual flow	21	Athlete's foot	74	Mouth bitter
	Less menstrual flow	22	Brittle nails	75	Saliva comes out
17	Irregular menstruation	23	Get tired easily	76	Throat pain
18	Delivery	24	Easy to sweat	77	Throat jams
19	Spontaneous abortion	25	Night sweats	78	Thirsty
20	Induced abortion	26	Hot flush	79	Dry mouth
21	Abnormal bleeding	27	Heat intolerance	80	Dry lips
22	Pregnancy toxemia	28	Cold intolerance	81	Take water often
		29	Attenuation of sexual desire	82	Tinnitus
		30	Impotence	83	Hearing loss
		31	Neck stiffness	84	Cough
		32	Shoulder stiffness	85	Asthma
		33	Back stiffness	86	Shortness of breath
		34	Lower back stiffness	87	Palpitation
		35	Facial pain	88	Chest pain
		36	Hand pain	89	Burp
		37	Foot pain	90	Heartburn
		38	Shoulder pain	91	Epigastric jamming discomfort
		39	Back pain	92	Nausea
		40	Hip pain	93	Vomiting
		41	Knee pain	94	Motion sickness
		42	Numbness face	95	Stomach fullness
		43	Numbness hands	96	Stomach rumbling
		44	Numbness legs	97	Flatulence
		45	Numbness back	98	Sleepy after eating
		46	Trembling face	99	Abdominal pain
		47	Trembling hands	100	Hand stiffness
		48	Trembling legs	101	Lower extremities weakness
		49	Hie general	102	Legs fluctuate
		50	Hie hands	103	Legs spasms
		51	Hie legs	104	Frostbite
		52	Hie lower back	105	Menstruation textile
		53	Heat face	106	Menstrual pain

We collected patients' subjective symptoms using a 128-item binary questionnaire. Of these symptoms, 106 corresponded to VAS questions when patients provided an affirmative response.

**Table 2:** Number of patients with *hie* (cold sensation) as per combination of body parts (%).

	Whole body (*n* = 617)	Hands (*n* = 859)	Legs (*n* = 1,797)	Lower back (*n* = 368)
Whole body	617 (26.3)			
Hands	172 (7.3)	859 (36.6)		
Legs	265 (11.3)	722 (30.8)	1,797 (76.7)	
Lower back	126 (5.4)	152 (6.5)	286 (12.2)	368 (15.7)

This table shows the combination of body parts where patients with *hie* (*n* = 2,344, including hiesho and non-hiesho groups) experienced their symptoms. As you can see, 30.8% felt *hie* in both their hands and legs/feet; that is, 84.2% of patients who felt *hie* in their hands also felt *hie* in legs/feet. Similarly, 12.2% felt *hie* in both their lower back and legs/feet; that is, 77.7% of patients who felt *hie* in their lower back also felt *hie* in legs/feet. In contrast, 11.3% of patients felt *hie* throughout their whole body and legs/feet; that is, 43% of patients who felt *hie* throughout whole body also felt *hie* in their legs/feet; this ratio was significantly lower than the former two.

**Table 3:** Ten most commonly associated symptoms for patients in the hiesho (cold disorder) group and frequencies in other groups.

	Hiesho (*n* = 524)	Non-hiesho (*n* = 1,820)	Normal (*n* = 1,672)
Mean number of accompanied symptoms ± SD	22.9 ± 1.0	24.5 ± 0.6	15.8 ± 0.5
Common symptoms (women [%]/men [%])			
Shoulder stiffness	338 (78.6)/56 (59.6)	1152 (77.9)/196 (57.3)	633 (63.5)/274 (40.6)
Easily fatigued	306 (71.2)/53 (56.4)	1071 (72.5)/212 (62.0)	562 (56.4)/311 (46.1)
Neck stiffness	283 (65.8)/43 (45.7)	970 (65.6)/168 (49.1)	466 (46.7)/200 (29.6)
Eyestrain	249 (57.9)/35 (37.2)	808 (54.7)/175 (51.2)	425 (42.6)/222 (32.9)
Depressed mood	186 (43.3)/31 (33.0)	721 (48.8)/139 (40.6)	338 (33.9)/183 (27.1)
Constipation	174 (40.5)/26 (27.7)	631 (42.7)/93 (27.2)	339 (34.0)/140 (20.7)
Upper back stiffness	172 (40.0)/25 (26.6)	577 (39.0)/98 (28.7)	240 (24.1)/84 (12.4)
Dry skin	164 (38.1)/27 (28.7)	624 (42.2)/140 (40.9)	334 (33.5)/247 (36.6)
Flatulence	154 (35.8)/35 (37.2)	510 (34.5)/135 (39.5)	247 (24.8)/162 (24.0)
Forgetfulness	145 (33.7)/42 (44.7)	499 (33.8)/124 (36.3)	283 (28.4)/153 (22.7)
Menstrual pain	156 (36.3)/0 (0.0)	580 (39.2)/0 (0.0)	239 (24.0)/0 (0.0)

The mean number of subjective symptoms from 122 symptoms for both hiesho and non-hiesho groups was significantly higher compared to normal group as shown by the *t*-test. We sorted symptoms by frequency in the hiesho group. The ranking of these symptoms was almost the same between the three groups and by participants' sex. Almost all symptoms were more common in the hiesho and non-hiesho groups compared to the normal group. In women, menstrual pain also was common. Results may be affected by the number of symptoms reported by patients.
